# Dissecting the opposing regulatory functions of endogenous nitric oxide production in colorectal cancer initiation, adaptive immune response alterations, and ferroptosis execution

**DOI:** 10.3389/or.2025.1671235

**Published:** 2025-11-05

**Authors:** Amol Tatode, Anis Ahmad Chaudhary, Mohammad Qutub, Rashmi Trivedi, Milind Umekar, Mohamed A. M. Ali, Tanvi Premchandani

**Affiliations:** ^1^ Division of Onco-Therapeutics, Department of Pharmaceutics, Smt. Kishoritai Bhoyar College of Pharmacy, Kamptee, Nagpur, Maharashtra, India; ^2^ Department of Biology, College of Science, Imam Mohammad Ibn Saud Islamic University (IMSIU), Riyadh, Saudi Arabia; ^3^ Department of Quality Assurance, Smt. Kishoritai Bhoyar College of Pharmacy, Kamptee, Nagpur, Maharashtra, India

**Keywords:** ferroptosis, angiogenesis, metabolic reprogramming, extracellular vesicles, oxidative stress, tumor microenvironment

## Abstract

Colorectal cancer (CRC) progresses through defined stages, from localized carcinoma *in situ* (Stage 0) to metastatic disease (Stage IV), with treatment strategies evolving from surgery in early stages to systemic therapies in advanced stages. Advances in biomarkers and genomic profiling have enabled personalized approaches, enhancing precision medicine. Nitric oxide (NO) plays a multifaceted role in CRC, acting as both a promoter and an inhibitor of cancer progression depending on its concentration, timing, and cellular context. At low concentrations, NO promotes angiogenesis, enabling tumor growth and metastasis. Conversely, high concentrations can exert anti-tumor effects, including the induction of cell death. Notably, its role in ferroptosis is biphasic: while high, exogenously delivered concentrations of NO can induce this iron-dependent cell death, lower, endogenously regulated levels can be protective by terminating lipid peroxidation. NO influences CRC by modulating the tumor microenvironment, mechanostress responses during metastasis, and signaling through extracellular vesicles (EVs), thereby aiding immune evasion. It also reprograms CRC cell metabolism, enhancing glucose utilization and mitochondrial activity to support growth in hypoxic conditions. The three nitric oxide synthases (NOS)—inducible NOS (iNOS), endothelial NOS (eNOS), and neuronal NOS (nNOS)—interact with hydrogen sulfide (H_2_S) to regulate oxidative stress and tumor growth. Targeting NO-related processes, such as ferroptosis, metabolic adaptations, and immune modulation, offers promising therapeutic advances to improve CRC treatment outcomes. This review highlights the dual role of NO in CRC, with particular focus on its novel mechanisms in ferroptosis, metabolism, immune modulation, and tumor–microenvironment interactions.

## Graphical Abstract

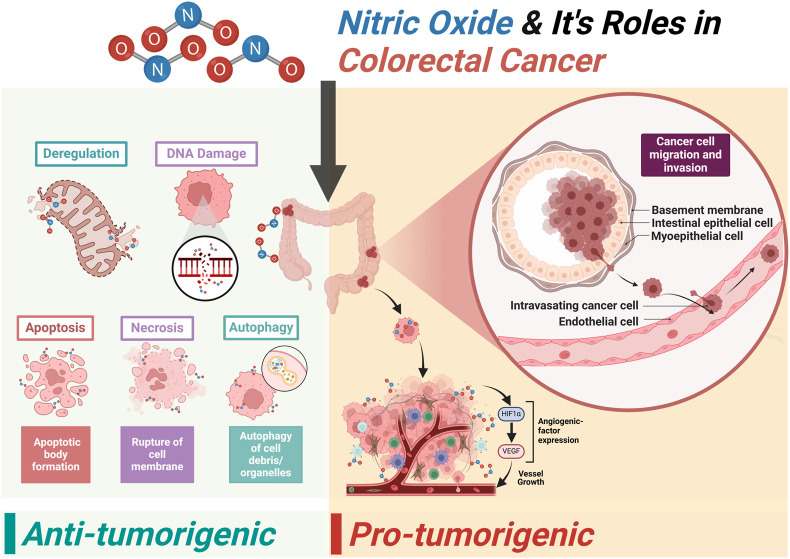

## Highlights

• Early screening reduces CRC mortality with accessible healthcare.
• Molecular oncology reveals CRC’s genetic and environmental complexities.
• NO modulates metastasis via mechanical force responses.
• Immune-cell-derived NO inhibits T-cell activity, enabling evasion.
• NO promotes angiogenesis, supporting tumor growth and survival.
• NO impacts gene expression, affecting apoptosis and DNA repair.
• NO–ROS interactions enhance CRC survival and chemoresistance.

## 1 Introduction

Colorectal cancer (CRC) is one of the most prevalent cancers globally (1). It originates in the colon or rectum, primarily from adenomatous polyps that may develop into malignancies over time (2, 3). Epidemiological data from 2023 reveal a shift in age-related trends, with CRC incidences increasing among younger populations (4, 5). Despite significant advancements in treatment, disparities in healthcare access continue to pose challenges in the management of CRC (6, 7). CRC progresses through well-defined stages, each reflecting the depth of tumor invasion, lymph node involvement, and distant metastasis (8, 9). The earliest stage, Stage 0 represents carcinoma *in situ*, with abnormal cells confined to the inner lining of the colon or rectum and minimal risk if treated early (10). In Stage I, the cancer invades the muscular layer but remains localized, without lymph node or distant spread (11). Stage II involves tumor extension through the colon wall, sometimes reaching adjacent organs, but lymph nodes remain unaffected, often requiring surgery and adjuvant therapies (12, 13). Stage III marks lymph node involvement, with systemic interventions such as chemotherapy becoming essential (14, 15). Stage IV is metastatic, with cancer spreading to distant organs such as the liver or lungs, necessitating complex therapies and palliative care (16, 17). Accurate staging is critical for personalized treatment planning (18, 19).

CRC remains a leading cause of cancer-related mortality worldwide, emphasizing the urgent need for effective treatment strategies (20–22). Advances in molecular oncology have revealed that a combination of genetic, environmental, and biochemical factors influences CRC progression. Among these, nitric oxide (NO) has gained attention due to its complex role in CRC development (23, 24). NO, a reactive molecule produced by nitric oxide synthase (NOS) enzymes, can either promote or inhibit cancer progression depending on its concentration, cellular location, and biochemical context (25, 26). Crucially, the biological effects of NO in CRC are not uniform; they are determined by a delicate balance of its concentration, cellular localization, tumor stage, and the surrounding redox environment. This review will dissect this dualism: at low, physiological concentrations, NO often exhibits pro-tumorigenic properties by promoting angiogenesis, supporting immune evasion, and preventing certain forms of cell death. Conversely, high, supraphysiological concentrations, often achieved through therapeutic delivery, typically exert anti-tumorigenic effects by inducing DNA damage, apoptosis, and ferroptosis. A central theme of this review is to delineate the context-dependent mechanisms that determine the functional role of NO. This dual nature of NO makes it both a challenging and promising target in CRC research, with potential applications across disease stages, from early tumor initiation to immune evasion and metastasis (27, 28). At low concentrations, NO can support tumor growth by facilitating angiogenesis, the process by which tumors form new blood vessels to sustain their nutrient and oxygen demands. This is particularly important for CRC as rapidly expanding tumors require an enhanced blood supply (29–31). NO contributes to this process by promoting the production of vascular endothelial growth factor (VEGF) and activating signaling pathways such as cGMP, both of which play roles in vascular development (32–34). In certain contexts, NO induces ferroptosis, although it can also inhibit this process depending on concentration and cellular state. In support of its ferroptosis-inducing role, recent evidence suggests that NO-releasing drugs, such as NCX4040, can directly trigger ferroptotic cell death in CRC cells. Specifically, NCX4040 treatment led to increased reactive oxygen species (ROS), lipid peroxidation, and activation of ferroptosis-related genes such as *CHAC1*, *GPX4*, and *NOX4*. These effects were reversed by ferrostatin-1, a specific inhibitor of ferroptosis, confirming the ferroptosis-dependent nature of NO-induced cell death in CRC models (35). Additionally, the development of self-catalyzing nitric oxide nanocomplexes has revealed another mechanism through which NO can promote ferroptosis (36). Moreover, combinations of NO with traditional ferroptosis inducers such as erastin have demonstrated synergistic effects, further supporting the pro-ferroptotic potential of NO-based therapies in CRC (35). Targeting this vulnerability could be valuable in CRC treatment by exploiting cancer cells’ sensitivity to oxidative damage. Thus, NO’s dual nature, with its tumor-promoting effects at low levels and cytotoxic effects at higher levels, presents both challenges and opportunities for CRC therapy (37–39). The therapeutic potential of targeting NO pathways in CRC has led to a variety of novel approaches (40). NOS inhibitors have shown promise in preclinical models by reducing CRC cell proliferation, angiogenesis, and immune suppression (41–43). Advances in drug delivery, such as NO-releasing nanoparticles, offer enhanced specificity by delivering therapeutic levels of NO directly to tumor sites, thus maximizing anti-tumor efficacy while minimizing off-target effects. These approaches underscore NO modulation as a potential strategy for addressing CRC’s therapeutic challenges (44–49).

This review examines NO’s role in CRC progression, highlighting its effects on molecular pathways, immune modulation, and metabolism. It explores NO’s influence on critical signaling pathways (Wnt/β-catenin, PI3K/AKT/mTOR, and NF-κB), immune modulation through pro-tumorigenic immune cell polarization, and metabolic reprogramming to sustain tumor growth and therapy resistance. The review also evaluates therapeutic strategies targeting NO, such as nitric oxide synthase inhibitors and NO delivery systems, while addressing challenges such as site-specific modulation and off-target effects.

## 2 Nitric oxide signaling networks in colorectal cancer

**FIGURE 1:**
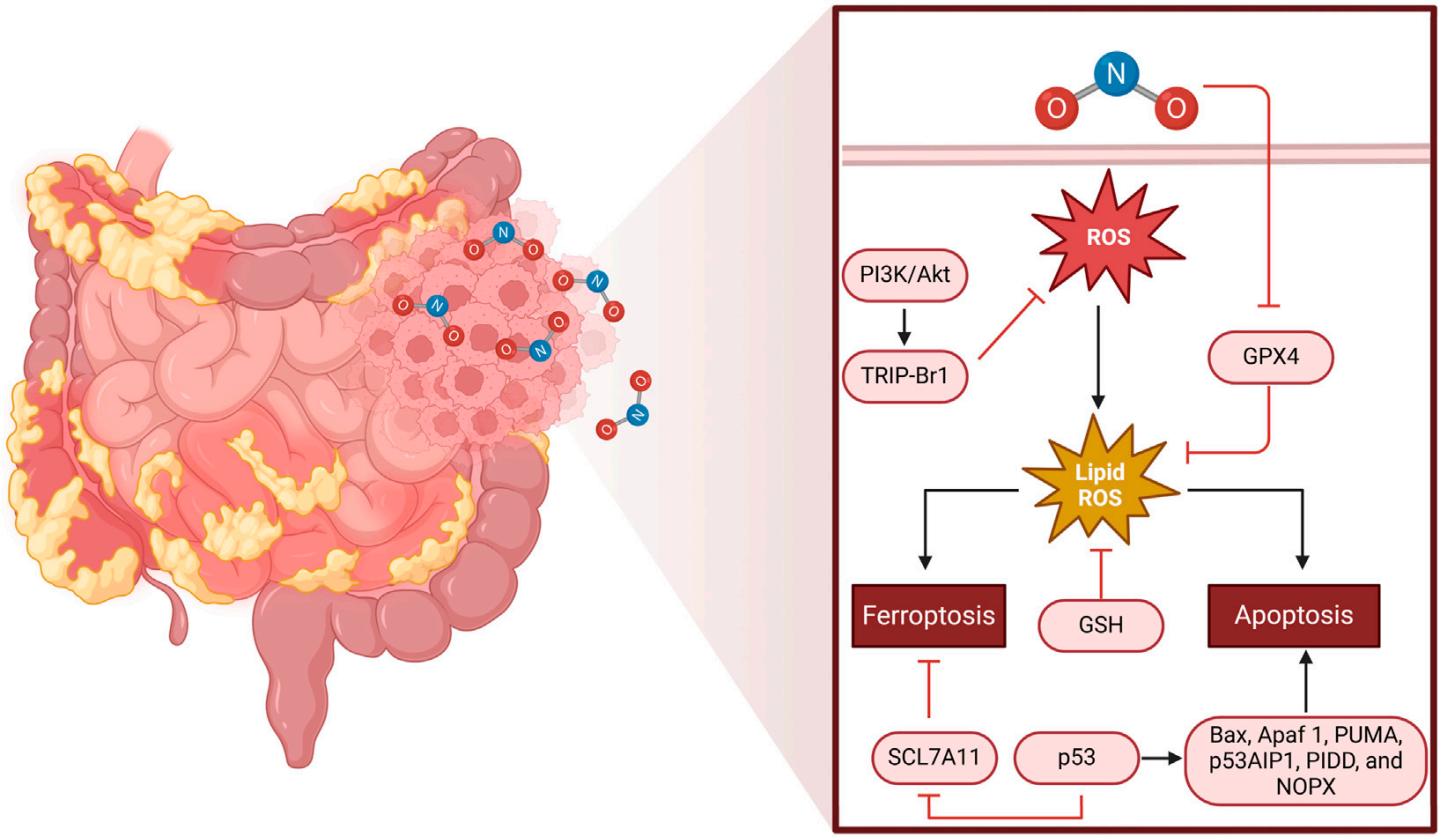
Mechanism of ROS-induced cell death in colorectal cancer. The left half of the figure depicts a portion of the human intestine, with emphasis on CRC tissue. The magnified area shows the molecular mechanisms of oxidative stress-induced cell death. NO triggers the production of ROS, which in turn causes lipid peroxidation (lipid ROS). This process can trigger ferroptosis through SCL7A11 inhibition and glutathione (GSH) loss or apoptosis through p53 activation and downstream targets (Bax, Apaf1, PUMA, p53AIP1, PIDD, and NOX). The PI3K/AKT pathway and TRIP-Br1 regulate ROS production, whereas GPX4 is an antioxidant protective mechanism against lipid peroxidation. The equilibrium among these pathways dictates CRC cell fate, either supporting survival or triggering programmed cell death.

In CRC, NO signaling has been implicated in multiple aspects of tumorigenesis, including tumor initiation, progression, metastasis, immune evasion, and therapeutic resistance (50, 51). The molecular and cellular mechanisms through which NO exerts its effects on CRC involve complex interactions among signaling pathways, redox biology, immune responses, and metabolic regulation (Figure 1; (52, 53)).

### 2.1 NO synthase isoforms and their roles in CRC

Each isoform plays a unique role in the pathophysiology of CRC, as mentioned in Table 1. Neuronal NOS (nNOS/NOS1) has emerging evidence suggesting a role in neuronal-like signaling within the tumor microenvironment (54). Expression of nNOS has been observed in certain CRC subtypes, where it may influence tumor cell migration and invasion (55, 56). Wang et al. (57) demonstrated that mtNOS1 suppresses cisplatin-induced mitochondrial superoxide accumulation and apoptosis in colon cancer cells by enhancing SIRT3 activity, which stabilizes SOD2—a key antioxidant enzyme. The mtNOS1–SIRT3–SOD2 axis underscores a mechanism through which cancer cells evade oxidative stress-driven death. Notably, geldanamycin, an Hsp90 inhibitor, blocked NOS1 mitochondrial translocation, reversing its anti-apoptotic effects and restoring chemosensitivity. These findings position mtNOS1 as an actionable target in overcoming therapy resistance. Expanding on this, Qiu et al. (58) revealed a novel link between hypercholesterolemia and NOS1-driven CRC progression. Their work showed that oxidized LDL (oxLDL) activates oxidant stress and hypoxia signaling, transcriptionally upregulating NOS1 in CRC cells. This pathway, mediated by the LOX-1 receptor, connects elevated cholesterol levels to enhanced NO production, fostering a tumor-permissive microenvironment. Crucially, pharmacological inhibition of NOS1 with Nω-propyl-L-arginine selectively curbed tumor growth in hypercholesterolemic models, suggesting a therapeutic strategy with reduced off-target toxicity. Together, these studies illuminate NOS1 as a dual regulator of chemoresistance and cholesterol-mediated tumorigenesis. The convergence of mitochondrial redox regulation (via SIRT3–SOD2) and hypercholesterolemia-induced hypoxia signaling on NOS1 highlights its centrality in CRC pathophysiology. Targeting NOS1 either through Hsp90 inhibitors to block its mitochondrial localization or via specific inhibitors such as Nω-propyl-L-arginine may offer dual benefits: mitigating therapy resistance and addressing metabolic risk factors in CRC (57, 58). Inducible NOS (iNOS/NOS2) is upregulated in response to pro-inflammatory cytokines such as tumor necrosis factor-alpha (TNF-α), interleukin-6 (IL-6), and interferon-gamma (IFN-γ). It is highly expressed in the inflammatory microenvironment of CRC and is associated with increased NO production, which promotes chronic inflammation, angiogenesis, and immune evasion (40, 59). NO generated by iNOS can induce DNA damage through the formation of peroxynitrite (ONOO^−^), a reactive nitrogen species that can cause mutagenesis and genomic instability, thus driving tumor progression (60).

**TABLE 1:** Roles of NOS isoforms in CRC: expression patterns, mechanisms, and therapeutic implications.

NOS isoform/aspect	Expression/change in CRC	Key findings/mechanisms	Reference
iNOS (NOS2)—early lesions	↑ in adenomas vs. normal; tends to be lower in advanced lesions	iNOS detected in colon adenomas; frequency decreases in carcinomas and is lowest in metastases. Association reported between iNOS and p53 alterations.	(65)
iNOS (NOS2)—EMT/metastasis	Often reduced in liver metastases vs. primary CRC	In paired primary–metastasis cohorts, NOS2 expression was lower in metastases; in experimental models, manipulating NOS2 in tumor cells did not robustly drive metastasis, suggesting context dependence.	(66)
eNOS (NOS3)	↑ in poor prognosis mesenchymal/stem-like CRC subtype	eNOS upregulated after Apc loss; functionally supports CSC/mesenchymal programs. Pharmacologic NO scavenging impaired mesenchymal/CSC traits in models.	(60)
iNOS–PARP-1 interaction/CSCs	PARP-1 signaling correlates with iNOS/NO effects in p53-dependent manner	In CRC patient cohorts and models, PARP-1 influenced CSC traits in a p53-dependent manner; iNOS-derived NO is considered a mediator linking PARP-1 to tumor progression.	(67)
iNOS and angiogenesis	↑ iNOS activity in tumors correlates with pro-angiogenic signaling	Tumor iNOS expression/activity correlates with VEGF, cGMP, microvessel density, and nitrotyrosine; higher iNOS is linked to the advanced stage.	(68)
Inflammation/IBD-associated carcinogenesis	iNOS/NT up in colitis and CAC lesions	Elevated iNOS and nitrotyrosine in colitis and human colon cancer; nitrative stress is implicated in inflammation-driven tumor initiation/progression.	(69)
Biphasic effects of NO	Low/steady NO: pro-tumor; high/sustained NO: cytotoxic	Classical dose/context-dependent paradigm: low NO supports growth/angiogenesis; high NO can trigger apoptosis and cytotoxicity.	(70) (176)
NOS uncoupling and oxidative stress	↓ BH4:BH2 → NOS uncoupling → O_2_-/peroxynitrite	The review highlights that insufficient BH4 leads to NOS uncoupling, which enhances ROS/RNS and pro-tumor signaling, thereby contributing to angiogenesis and therapy resistance pathways.	(50)
iNOS and apoptosis regulation	High NO promotes apoptosis; low NO can be pro-survival	Mechanisms include p53 activation, mitochondrial damage/cytochrome-c release, and caspase signaling at high NO; conversely, lower NO can nitrosylate and inhibit caspases or support survival pathways.	(70, 71)
iNOS inhibition and chemoprevention	NOS inhibitors show antitumor activity in preclinical studies	Reviews summarize preclinical efficacy of NOS inhibition (e.g., L-NAME, 1,400 W, and others) against tumor growth/angiogenesis; clinical translation remains challenging.	(72)

Endothelial NOS (eNOS/NOS3) is primarily expressed in endothelial cells, where it regulates vascular tone and angiogenesis (61). In CRC, eNOS expression is often elevated in tumor-associated endothelial cells, contributing to the formation of an abnormal vasculature that supports tumor growth. However, eNOS can also have tumor-suppressive effects, particularly through the generation of low levels of NO, which can promote apoptosis and enhance immune surveillance (62). Garza Treviño et al. (63) and Lu et al. (64) indicated that eNOS is upregulated in human mesenchymal CRC tumors and may represent an active stem-cell regulatory point in cancer and a possible target for therapy against aggressive human tumors (63, 64).

### 2.2 NO and immune modulation in CRC

Nitric oxide functions as a double-edged mediator in CRC. Depending on its source, concentration, and the surrounding environment, it can either promote or inhibit tumor growth. Its main function involves interacting with key immune cells such as tumor-associated macrophages (TAMs), myeloid-derived suppressor cells (MDSCs), and cancer-killing cytotoxic T lymphocytes (CTLs).

A major strategy to combat CRC involves targeting arginine metabolism to reactivate the immune system. For example, engineered microparticles can be used to inhibit the enzyme arginase while activating NOS. This cleverly shifts tumor-friendly M2-like TAMs into pro-inflammatory, cancer-fighting M1 types. This switch enhances endogenous NO production, which synergizes with photodynamic therapy to create a hostile tumor environment, leading to significant regression in resistant CRC models (73). Similarly, low doses of the PARP inhibitor olaparib can reduce the immunosuppressive activity of MDSCs by downregulating arginase-1 (ARG1) and iNOS, thereby restoring the activity of CTLs. When combined with anti-PD-1 therapy, this approach completely eradicated microsatellite instability-high (MSI-high) tumors and significantly reduced the size of microsatellite stable (MSS) tumors (74). These findings underscore the therapeutic potential of modulating arginine metabolism to enhance the efficacy of immune checkpoint therapies.

The tumor microenvironment (TME) often functions as a barrier that restricts immune cells infiltration. However, these barriers can be overcome. Nanoemulsions delivering melittin and NO donors can reverse the activation of cancer-associated fibroblasts (CAFs), reduce the dense collagen that shields the tumor, and normalize blood vessels, thereby facilitating CTL infiltration. When combined with anti-CTLA-4 therapy, this remodeling approach effectively suppressed tumor growth in CRC models rich in CAFs (75). Another approach involves using all-trans retinoic acid (AtRA) to suppress signaling pathways such as TLR4/NF-κB, which reduces the expression of iNOS and TNF-α in colitis-associated CRC (76). These strategies highlight the importance of targeting the tumor’s supportive structures to overcome immune exclusion.

Natural phytochemicals also show great promise. Phloretin, for example, has powerful anti-inflammatory effects, reducing NO and ROS levels in co-cultures of cancer and immune cells. It works by inhibiting NF-κB, which helps protect the gut lining and reduces inflammatory signals (77). Extracts from organisms such as Micractinium sp. can also suppress key inflammatory enzymes such as COX-2 and iNOS in macrophages while halting the cancer cell cycle (78). Combining different therapies is also incredibly effective. Angiotensin II receptor blockers (ARBs) can disrupt MDSC function, which boosts anti-PD-L1 activity and substantially increases the number of tumor-specific CD8^+^ T-cells (79). In another study, the combination of cyclophosphamide with Toll-like receptor agonists (TLRAs) eliminated MDSCs and activated tumoricidal myeloid cells, leading to complete tumor regression in an NO-dependent manner (80). Even in clinical settings, combining cetuximab with chemotherapy reduces plasma iNOS and NO levels in patients with metastatic CRC, which correlates with better T-cell responses (81).

Although these preclinical studies are promising, translating them into clinical practice requires caution. It is important to account for different tumor subtypes and determine the appropriate dosing to balance the beneficial and detrimental effects of NO as excessive NO can promote DNA damage and tumor growth. Future research needs to focus on finding biomarkers, such as ARG1/iNOS expression or TAM polarization status, to predict which patients are most likely to benefit. The ultimate goal is to develop personalized combinations of NO modulators and checkpoint inhibitors. A deep and nuanced understanding of the context-dependent roles of NO is essential to fully unlock its therapeutic potential and advance treatment for CRC.

### 2.3 NO-mediated ferroptosis in CRC

The relationship between NO and ferroptosis in CRC represents a critical and complex paradox. Initially regarded as a straightforward pro-ferroptotic agent due to its role in generating reactive nitrogen species (RNS), emerging evidence reveals a potent, context-dependent anti-ferroptotic function. Understanding this duality is essential as endogenous NO within the tumor microenvironment may protect cancer cells from ferroptotic death, while exogenous, high-dose NO delivery represents a promising therapeutic strategy to forcibly induce it.

At high or bolus concentrations, typically achieved with therapeutic NO-donors, NO strongly promotes ferroptosis through several interconnected mechanisms. The primary pathway involves its rapid reaction with superoxide radicals (O_2_⋅−) to form peroxynitrite (ONOO−), a highly potent and damaging oxidant. This potent oxidant induces ferroptosis by depleting glutathione (GSH), the cell’s principal soluble antioxidant, thereby compromising the primary defense against lipid peroxides. This effect is further compounded by the inactivation of glutathione peroxidase 4 (GPX4) as ONOO− can modify the selenocysteine residue within its active site, thereby disabling the only enzyme capable of directly reducing complex lipid hydroperoxides in biological membranes. The resulting accumulation of lipid ROS, coupled with the ability of ONOO− to directly initiate lipid peroxidation, creates a vicious cycle of membrane damage. Furthermore, NO can modulate iron homeostasis by promoting the release of iron from ferritin, which increases the labile iron pool (Fe^2+^) available to catalyze the Fenton reaction and amplify lipid peroxidation. This pro-ferroptotic role of NO provides the rationale for using NO-releasing drugs to induce ferroptotic cell death in CRC (Tables 2, 3).

**TABLE 2:** Nitric oxide-mediated modulation of ferroptosis: pro- and anti-ferroptotic mechanisms across various study models.

Effect	Study model	NO source/manipulation (concentration where reported)	Ferroptosis markers/readouts reported	Main outcome (accurate, from the paper)	DOI/reference
Pro-ferroptotic	Immortalized mouse hippocampal HT22 cells treated with sulfasalazine (SAS)	SAS → PDI-mediated iNOS dimerization and ↑ cellular NO (the study reports iNOS upregulation and NO accumulation; manipulation: SAS treatment)	↑ cellular NO, ↑ ROS, and ↑ lipid-ROS; cell death with ferroptotic features	SAS induced ferroptotic death in HT22 accompanied by PDI-dependent iNOS dimerization → NO accumulation and lipid-ROS; PDI inhibition or knockdown suppressed iNOS dimerization, NO/lipid-ROS accumulation and protected cells. (i.e., NO production via iNOS is required in this model).	(85)
Pro-ferroptotic	*In vivo*—motor cortex after spinal cord injury (rat + human tissue correlates)	Activated microglia secrete abundant NO (microglial iNOS induction described)	Iron overload measures, ↑ lipid ROS, MDA, and altered ferroptosis-related gene expression	Microglial activation → NO release that dysregulates iron homeostasis; iron overload in motor cortex triggered lipid-ROS accumulation and neuronal ferroptosis after SCI (the study links microglial NO to iron handling dysfunction, leading to ferroptosis).	(86)
Pro-ferroptotic	*In vitro* (ovarian cancer cell lines), plus mouse xenografts	Sodium molybdate treatment → ↑ NO production (the study states that NO mediates GSH depletion)	↑ labile iron pool (LIP), ↓ GSH, and ↑ lipid peroxidation (4-HNE) and markers of ferroptosis; mitochondrial dysfunction readouts for apoptosis were also reported	Sodium molybdate increases LIP and induces NO production, leading to GSH depletion and ferroptosis in ovarian cancer cells; it also produces mitochondrial damage and apoptosis *in vivo*.	(87)
Pro-/context-dependent	*In vitro* (β-cells/pancreatic islets)—cytokine exposure models/reviews of β-cell death	Cytokine-induced iNOS/NO production (studies used cytokines; iNOS inhibitors were also used to probe its role)	Lipid peroxidation/GPX4 and GSH measurements; outcome assessed using ferroptosis inhibitors	Several papers show that in cytokine-exposed β-cells, endogenous NO produced via iNOS prevents ferroptosis (favoring apoptosis instead); conversely, blocking NO production can increase lipid peroxidation in that system. Conclusion: NO can be anti-ferroptotic in cytokine-stressed β-cells —not pro-ferroptotic (the original entry asserting that NO exacerbates ferroptosis in β-cells is not supported; the primary study shows that NO derived from iNOS protects β-cells from ferroptosis).	(88)
Pro-ferroptotic (when eNOS inhibited)	*In vitro* H9c2 cardiomyocytes or OGD/R H9c2 (cardiomyocyte) model	miR-199a-5p overexpression → inhibition of AKT/eNOS signaling → reduced eNOS activity/NO	↑ ROS, ↑ MDA, ↑ Fe^2+^, ↓ GSH/GSSG ratio, and ↓ GPX4	In H9c2 models of ischemia/reoxygenation, miR-199a-5p promoted ferroptosis by inhibiting AKT/eNOS signaling (i.e., loss of eNOS/NO signaling is associated with enhanced ferroptosis markers). So, decreased eNOS/NO is pro-ferroptotic.	(89)
Anti-ferroptotic	*In vitro* Hepa1-6 (mouse hepatoma) and other cell lines	NOC18 (long-lived NO donor) pre-incubation/NO donors	↓ C11-BODIPY lipid peroxidation signal and ↓ 4-HNE; GPX4 and GSH quantified	NOC-18 and other NO donors terminate the lipid-peroxidation chain reaction and protect against ferroptosis induced by cysteine starvation, GPX4 inhibition, or TBHP (strong experimental evidence reveals that NO can be protective by terminating lipid peroxidation).	(82)
Anti-ferroptotic	*In vitro*/co-culture models (macrophages/epithelial cells) and infection models	iNOS in M1 macrophages → macrophage NO production (physiological iNOS induction)	↓ phospholipid-peroxide signals (15-HpETE-PE) and ↓ lipid peroxidation markers; rescue assays with inhibitors	Macrophage iNOS/NO acts as an inter-cellular anti-ferroptotic mechanism: macrophage-derived NO prevents phospholipid peroxidation (notably 15-HpETE-PE) in adjacent epithelial cells and protects cells from ferroptosis (e.g., during *Pseudomonas* infection). This is a clear anti-ferroptotic mechanism.	(90)
Anti-ferroptotic (drug model)	*In vivo* rat traumatic brain injury (TBI) model	Propofol administered IP (30 mg/kg)—propofol ↑ eNOS expression and NO content	↑ GPX4 (protein), ↓ 4-HNE/lipid peroxidation, and ↓ iron deposition; behavioral/cognitive readouts improved	Propofol improved TBI outcomes and decreased ferroptosis markers; mechanistic data show that propofol increases eNOS and NO and the protective effect is reversed by eNOS inhibitor L-NAME → supports eNOS/NO axis as anti-ferroptotic in this TBI model.	(91)
Anti-ferroptotic/PDI inhibition	*In vitro* HT22 cells—catechol estrogen study	Catechol estrogens (PDI inhibitors) prevent PDI-mediated NOS activation	↓ lipid-ROS and protection from erastin/SAS-induced ferroptosis	Catechol estrogens inhibit PDI catalytic activity, reducing PDI-mediated NOS activation and protecting HT22 cells from chemically induced ferroptosis (supports the mechanistic role of PDI→NOS→NO→lipid-ROS in certain models).	(92)
PAL/plasma-activated Ringer’s lactate (contextual)	*In vitro* malignant mesothelioma (MM) cells; PAL exposure	PAL increases citrulline–NO cycle activity → iNOS induction; lysosomal NO accumulation	↑ lipid peroxidation, lysosomal peroxynitrite, and iNOS upregulation; ferroptosis indicators	PAL (plasma-activated Ringer’s) induces ferroptosis in MM cells, with NO/iNOS playing a central role (lysosomal NO/peroxynitrite contributes to lipid peroxidation and ferroptosis); inhibition of NO or iNOS reduced PAL-induced ferroptosis. Context is PAL exposure—NO here contributes to ferroptosis.	(91)

**TABLE 3:** Ferroptosis in colorectal cancer and the role of nitric oxide.

Model/condition	Method/timepoint	Key finding (↑/↓)	Mechanistic insight	Therapeutic implication	NOS effect/relation	Reference
NCX4040 in HT-29 and HCT116 cells	MitoSox assay (4 h); MDA assay (2–4 h); RT-PCR (4 and 24 h); metabolomics (24 h)	NCX4040 treatment → ↑ ROS, ↑ lipid peroxidation, ↑ CHAC1, ↑ GPX4, and ↑ NOX4; cell viability ↓; combination with erastin/RSL3 further ↓ viability; ferrostatin-1 preserves viability	NCX4040 (a nitric oxide donor) triggers ferroptosis via oxidative stress and lipid peroxidation	Combining NCX4040 with other ferroptosis inducers may overcome chemoresistance	Direct: NCX4040 is a NO-donor releasing NO to initiate the ferroptotic cascade	(35)
NOS2 in clinical CRC and NOS2-overexpressing CRC cells	Western blot, qRT-PCR, and xenograft assays	NOS2 expression ↑ in tumors; NOS2 overexpression → NF-κB signaling ↓, GPX4 ↓, and tumor growth ↓	NOS2 modulates inflammatory and antioxidant pathways, promoting ferroptosis	NOS2 may serve as a prognostic marker and therapeutic target	Direct: NOS2-derived NO modulates NF-κB and GPX4, thereby promoting ferroptosis	(93)
CRC cells + ferroptosis modulators	Cytotoxicity and gene expression assays	Treatment → ↑ lipid peroxidation; SLC7A11 expression ↓; cell viability ↓	Inhibition of cystine uptake (via SLC7A11) drives ferroptosis	Targeting SLC7A11 could overcome drug resistance in CRC	Direct: NO signaling can modulate oxidative stress that influences SLC7A11 expression	(94)
CRC cells + RSL3	ROS and viability assays	RSL3 inactivates GPX4 → ↑ ROS and ↑ lipid peroxidation; cell viability ↓	Direct inhibition of GPX4 triggers ferroptosis	GPX4 is a key target for ferroptosis induction in cancer cells	Indirect: basal NO levels may influence redox balance, although NOS is not directly addressed	(95)
KRAS-mutant CRC + Cetuximab + RSL3	Viability and signaling assays (24 h)	Cetuximab → Nrf2/HO-1 signaling ↓; combined with RSL3 → ↑ ferroptosis; cell viability ↓	Combination therapy downregulates antioxidant defenses, enhancing ferroptosis	May overcome resistance in KRAS-mutant CRC	Indirect: while not modulating NOS directly, NO can influence Nrf2/HO-1 and related oxidative pathways	(96)
CRC cells + propofol	STAT3 expression and viability assays	Propofol → STAT3 expression ↓; ferroptosis ↑; cell viability ↓	Downregulation of STAT3 facilitates ferroptosis in CRC cells	Propofol may sensitize CRC cells to ferroptosis-inducing therapies	Indirect: propofol’s modulation of STAT3 can be affected by NO bioavailability in the microenvironment	(97)
CRC cells + apatinib	Signaling pathway analysis and viability assays	Apatinib modulates ELOVL6/ACSL4 signaling → ↑ ferroptosis; cell viability ↓	Activation of lipid metabolism (ELOVL6/ACSL4) promotes ferroptosis	Apatinib is a potential ferroptosis inducer in CRC therapy	Indirect: NO can modulate lipid peroxidation; thus, NO signaling may complement apatinib’s effects	(98)
CRC cells + talaroconvolutin A	Lipid peroxidation and viability assays	Talaroconvolutin A → ↑ lipid peroxidation and ↑ ferroptosis; cell viability ↓	Enhances lipid peroxidation to drive ferroptosis	Could reverse drug resistance by triggering ferroptosis	Indirect: NO-mediated oxidative stress may amplify lipid peroxidation induced by talaroconvolutin A	(99)
CRC cells + ginsenoside Rh3	Analysis of STAT3/p53/NRF2 axis	Rh3 → STAT3 expression ↓; modulation of p53/NRF2 → ↑ ferroptosis; cell viability ↓	Modulation of tumor suppressor pathways (p53/NRF2) induces ferroptosis	May be used as an adjunct to improve ferroptosis-based therapies	Indirect: alterations in these pathways can be modulated by NO signaling, impacting ferroptosis	(100)
p53 activation in cancer cells	p53 activation assays	p53 activation → cystine uptake ↓, resulting in ↑ ferroptosis	p53 downregulates SLC7A11, thereby promoting ferroptosis	Exploiting p53 pathways may trigger ferroptosis in tumors	Indirect: p53 activity can be modulated by NO, influencing SLC7A11 regulation	(101)
CRC cells + andrographolide ± 5-FU	Viability and ferroptosis assays	Andrographolide → ↑ ferroptosis; synergy with 5-FU → cell viability ↓	Induction of ferroptosis enhances chemosensitivity	Andrographolide could serve as an adjuvant with 5-FU in CRC treatment	Indirect: andrographolide can affect NF-κB and related pathways, which are sensitive to NO levels	(102)
CRC cells + BSO	GSH depletion, ROS measurement, and viability assay	BSO → GSH ↓, ↑ ROS, triggering ↑ ferroptosis; cell viability ↓	GSH depletion compromises antioxidant defenses, leading to ferroptosis	BSO may sensitize CRC cells to ferroptosis-inducing agents	Indirect: NO can further exacerbate oxidative stress when GSH is depleted	(103)
CRC cells with GPX4 Inhibition	Lipid peroxidation and viability assays	GPX4 inhibition → ↑ lipid peroxidation, ↑ ferroptosis; cell viability ↓	Direct targeting of GPX4 disrupts redox homeostasis, triggering ferroptosis	GPX4 is a prime target for inducing ferroptosis in CRC	Indirect: Basal NO levels may enhance oxidative damage when GPX4 is inhibited	(104)
CRC cells + miR-15a-3p overexpression	miRNA transfection, GPX4 measurement, viability assay	miR-15a-3p overexpression → GPX4 ↓, resulting in ↑ ferroptosis; cell viability ↓	miR-15a-3p suppresses GPX4 expression, promoting ferroptosis	miR-15a-3p may be developed as a therapeutic tool to induce ferroptosis	Indirect: NO signaling can influence GPX4 regulation; thus, miR-15a-3p effects might be modulated by NO	(105)
CRC cells + dihydroartemisinin (DHA)	Labile iron pool and ROS assays, viability assay	DHA → labile iron pool (LIP) ↑ and ROS ↑, triggering ↑ ferroptosis; cell viability ↓	DHA increases intracellular iron and ROS, thereby driving ferroptosis	DHA is a potential ferroptosis inducer for CRC therapy	Indirect: NO can synergize with ROS, amplifying ferroptosis induced by increased iron levels	(106)
CRC cells + miR-148a-3p overexpression	miRNA assay targeting SLC7A11 and viability assay	miR-148a-3p overexpression → SLC7A11 ↓, leading to ↑ ferroptosis; cell viability ↓	Downregulation of SLC7A11 via miR-148a-3p facilitates ferroptosis	miR-148a-3p holds promise as a prognostic and therapeutic target in CRC	Indirect: NO-related oxidative stress may further decrease SLC7A11 expression, enhancing miR-148a-3p effects	(107)

Conversely, at lower, physiologically relevant concentrations, often produced endogenously by NOS enzymes, NO can act as a powerful inhibitor of ferroptosis. This protective function is primarily attributed to its intrinsic properties as a radical species. As demonstrated compellingly by Homma et al. (82), NO can directly react with and terminate lipid peroxyl radicals (LOO⋅), effectively acting as a chain-breaking antioxidant that inhibits the propagation of lipid peroxidation and protects cells from ferroptosis induced by GPX4 inhibition or cysteine starvation. Beyond this direct chemical defense, NO also functions as a critical signaling molecule, modulating protein function through regulatory S-nitrosylation to actively suppress the ferroptotic cascade. For instance, NOS1-mediated S-nitrosylation of PTEN leads to its degradation and subsequent activation of the pro-survival AKT/mTOR pathway, which can suppress autophagy-dependent ferroptosis (82, 83).

Moreover, this ferroptosis-suppressive effect appears to be conserved *in vivo*. NOS2 knockout mice exhibit enhanced inflammation and lipid peroxidation under stress, supporting the idea that endogenous NO production has cytoprotective, anti-ferroptotic effects in mammalian tissues (84). Additionally, the microbiome’s role in modulating NO–ferroptosis crosstalk via microbial metabolites influencing iron availability or ROS levels remains largely unexplored, although recent studies have begun to elucidate this topic. For example, gut microbiota metabolites such as short-chain fatty acids (SCFAs) and tryptophan derivatives indirectly affect ferroptosis by regulating oxidative stress and iron metabolism. SCFAs can upregulate NLRP6 expression and promote RIG-I/MAVS-mediated mitophagy, thereby inhibiting ferroptosis. Tryptophan metabolites such as kynurenine (KYN) can scavenge ROS and activate the Nrf2-dependent pathway to regulate cellular ferroptosis. Although not extensively studied, these findings suggest that the gut microbiota may influence NO–ferroptosis crosstalk through metabolic pathways, warranting further investigation.

The switch between NO’s pro- and anti-ferroptotic roles is determined by a delicate interplay of concentration and the local redox environment. In a high-oxidative-stress environment rich in superoxide, NO is rapidly converted to pro-ferroptotic ONOO −. In contrast, when superoxide levels are lower, NO can persist and exert its anti-ferroptotic, radical-scavenging effects. Therefore, the low, sustained levels of NO often found in the CRC tumor microenvironment may confer a survival advantage by protecting cancer cells against ferroptotic stress. This presents a therapeutic challenge as endogenous NO could contribute to resistance against ferroptosis-inducing chemotherapies. However, it also highlights a therapeutic opportunity: overwhelming this protective system with high, localized doses delivered via NO-releasing nanocarriers can trigger ferroptotic cell death in CRC cells (Table 3). This biphasic nature positions the NO signaling pathway as a sophisticated, druggable node in the regulation of CRC cell death.

The seemingly contradictory roles of NO in ferroptosis, as detailed in Tables 2, 3, can be reconciled by considering several key factors. The balance between pro- and anti-ferroptotic outcomes is largely dictated by the net effect of NO on the cellular redox state, specifically the ROS/GSH balance, and its targeted S-nitrosylation of key regulatory proteins. At high concentrations, NO can react with superoxide to form peroxynitrite (ONOO^−^), a potent oxidant that depletes GSH and initiates lipid peroxidation, thereby driving ferroptosis. Conversely, at lower, controlled concentrations, NO can act as a radical-trapping antioxidant, directly terminating lipid peroxidation chain reactions and preventing the accumulation of toxic lipid peroxides. Furthermore, S-nitrosylation of specific targets can either promote (e.g., inhibiting GPX4) or inhibit (e.g., activating survival pathways) ferroptosis. Therefore, the ultimate effect of NO is not absolute but is instead a product of its concentration, the local redox environment, and the specific molecular machinery of the target cell.

## 3 Interaction between NO and H_2_S in CRC

The interplay between NO and hydrogen sulfide (H_2_S) constitutes a dynamic regulatory axis in CRC, with profound implications for redox balance, therapeutic resistance, and immune evasion. Mechanistically, their crosstalk is bidirectional and often antagonistic. These gasotransmitters engage in reciprocal post-translational modifications of NO via S-nitrosylation and H_2_S via S-sulfhydration to modulate critical signaling pathways. For instance, NO suppresses cystathionine β-synthase (CBS), the primary H_2_S-producing enzyme, thereby reducing endogenous H_2_S levels and impairing tumor survival (108). Conversely, H_2_S inhibits endothelial nitric oxide synthase (eNOS), curtailing NO bioavailability and altering vascular dynamics. This antagonistic relationship extends to mitochondrial function H_2_S scavenges ONOO^−^, a cytotoxic reactive nitrogen species, an effect that can be mitigated by H_2_S, thereby creating a complex regulatory loop that governs mitochondrial dysfunction and redox balance (109). These interactions underscore the delicate redox equilibrium that governs CRC progression.

In multidrug-resistant CRC, the NO–H_2_S axis emerges as a therapeutic vulnerability. Co-treatment with NO and H_2_S donors downregulates P-glycoprotein (MDR1), reducing chemotherapeutic efflux and resensitizing tumors to conventional agents. This synergy highlights their potential to circumvent resistance mechanisms rooted in drug transporter overexpression. Furthermore, the TME dictates context-specific effects. It has been experimentally demonstrated that NO, particularly when produced endogenously under hypoxic conditions, can stabilize hypoxia-inducible factor-1α (HIF-1α). In colon carcinoma HCT116 cells, Chowdhury et al. (110) showed that endogenously generated NO and ROS contribute to HIF-1α accumulation by inhibiting prolyl hydroxylase domain proteins (PHDs) via S-nitrosation of PHD2, thus preventing HIF-1α degradation during hypoxia (110). Further supporting this, Lee et al. (111) demonstrated that in Caco-2 epithelial cells, nitric oxide produced during exposure to *Clostridium difficile* toxin facilitated HIF-1α stabilization through iNOS-dependent S-nitrosylation. Inhibition of iNOS reduced HIF-1α accumulation and worsened epithelial damage, emphasizing NO’s protective role via this pathway (111–113).

These platforms exploit the pro-death effects of gasotransmitter fluxes, achieving localized redox disruption without compromising healthy tissues. Immunologically, the NO–H_2_S axis modulates dendritic cell activation and suppresses MDSC expansion, thereby restoring antitumor immunity. This immunomodulatory role, coupled with their ability to induce ferroptosis, provides a rationale for combining gasotransmitter-targeted therapies with immune checkpoint inhibitors. Future investigations should unravel the epigenetic implications of NO–H_2_S crosstalk, particularly their influence on histone acetylation and DNA methylation patterns that drive CRC aggressiveness. Additionally, spatial mapping of gasotransmitter fluxes within the TME, coupled with single-cell analyses, could identify niche-specific vulnerabilities. The development of dual NO/H_2_S modulators, capable of fine-tuning their synergistic or antagonistic effects, represents a frontier in precision oncology.

A significant gap in the current understanding is the influence of the gut microbiome on this crosstalk as microbial metabolites could modulate the local availability of both gasotransmitters and their precursors, thereby influencing CRC progression. Furthermore, developing dual NO/H_2_S modulators capable of fine-tuning their synergistic or antagonistic effects represents a promising frontier in precision oncology.

### 3.1 Molecular crosstalk between NO and H_2_S

NO plays a multifaceted role in CRC, influencing tumor progression, angiogenesis, and therapeutic responses through diverse molecular mechanisms. Recent studies have demonstrated that uncoupled NOS activity contributes to CRC progression by generating reactive oxygen/nitrogen species (ROS/RNS). Alam et al. (50) reported that sepiapterin, a tetrahydrobiopterin precursor, recoupled NOS in CRC cell lines (HCT116 and HT29), restoring the tetrahydrobiopterin: dihydrobiopterin ratio, which is significantly lower in tumors than in normal tissues. This intervention reduced proliferation and induced apoptosis via AKT/GSK-3β-mediated β-catenin downregulation. In murine models, oral sepiapterin decreased metabolic uptake of fluorodeoxyglucose and significantly increased apoptosis in azoxymethane/dextran sodium sulfate-induced CRC tumors. Extracellular vesicles (EVs) derived from CRC cells further amplify NO-mediated pathways. Ikeda et al. identified CAT1-positive EVs in CRC patients, which enhanced arginine transport and NO synthesis in endothelial cells, promoting angiogenesis. Plasma EV-CAT1 levels were significantly elevated in CRC patients, correlating with increased vascular endothelial cell growth and tubule formation (115).

Immunomodulatory strategies targeting NO pathways have shown therapeutic promise. (116) engineered exosomes (exoASO-STAT6) to silence STAT6 in TAMs, inducing M1 polarization through NOS2 upregulation. This approach generated a substantial amount of NO in triple-negative breast and CRC cells, leading to significant tumor growth inhibition and a high rate of complete remission in syngeneic CRC models. Ferroptosis induction via NO donors has also been explored. (35) demonstrated that NCX4040, a non-steroidal NO donor, generated ROS in CRC cells (HT29 and HCT116) without glutathione depletion. Co-treatment with ferroptosis inducers (erastin or RSL3) synergistically enhanced cell death, while ferrostatin-1, a ferroptosis inhibitor, markedly reduced cytotoxicity. Lipid peroxidation increased dose-dependently, and metabolomic profiling revealed upregulated expression of CHAC1, GPX4, and NOX4, key regulators of ferroptosis. Real-time NO detection methodologies have further advanced therapeutic targeting. Daw et al. (117) developed an oxyhemoglobin-based assay quantifying NO production in cytokine-stimulated CRC cells. IFN-γ, IL1-β, and TNF-α induced NOS2 expression, producing a quantifiable amount of NO. The NOS2 inhibitor 1,400 W exhibited a potent IC_50_ value in 4T1 cells, with rapid inhibition that persisted for an extended period.

H_2_S, another gaseous signaling molecule, has been implicated in CRC vascular function, although research remains limited. Hassan et al. investigated the vasodilatory effects of NO and H_2_S in human mesenteric arteries obtained from CRC patients. Sodium nitroprusside (SNP)-induced NO-mediated relaxation was significantly reduced by tetraethylammonium (TEA), a K+ channel blocker, compared to controls. H_2_S-induced vasorelaxation involved KATP channels, although its specific role in CRC progression remains underexplored. The study highlighted that H_2_S and NO interactions may regulate vascular tone in CRC, with potential implications for tumor microenvironment modulation (118).

NO exerts profound effects on CRC through angiogenesis, immunomodulation, and ferroptosis, with therapeutic strategies targeting NOS coupling, EV-mediated pathways, and ferroptosis induction showing significant promise. H_2_S, while less studied, appears to modulate vascular function in CRC, warranting further investigation. Translating these preclinical findings into clinical applications requires validation of exact molecular mechanisms and dose-response relationships, particularly for H_2_S, to develop targeted therapies for CRC.

## 4 Novel therapeutic approaches of NO signaling in CRC

**FIGURE 2:**
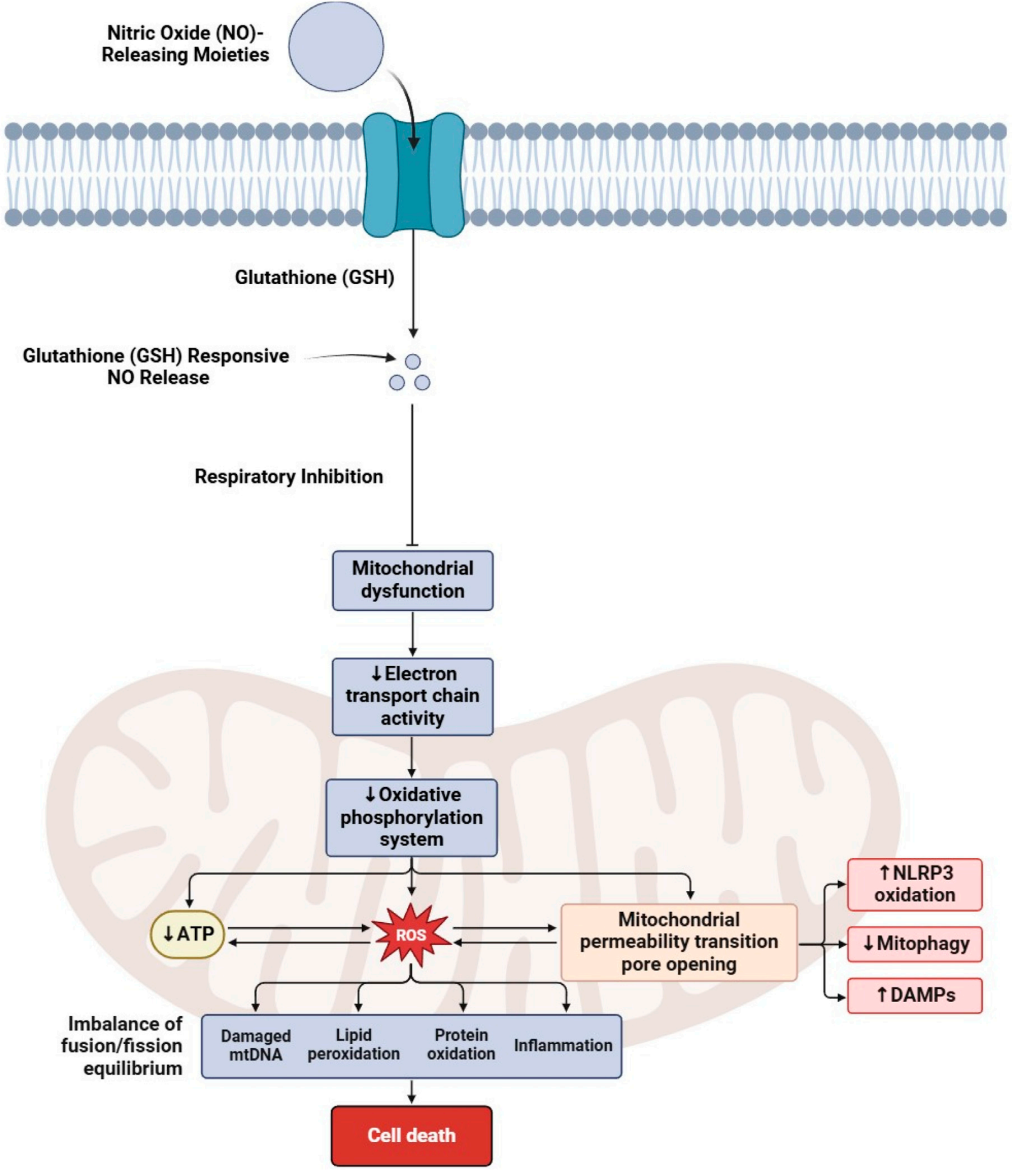
Mechanism of nitric oxide-induced mitochondrial dysfunction leading to cell death.

The therapeutic potential of targeting NO signaling and its associated molecular pathways has attracted significant attention in CRC research in recent years (119, 120). Several studies have explored various aspects of NO signaling in CRC, including its impact on ferroptosis, mitochondrial dysfunction (Figure 2), angiogenesis, immune regulation, and metabolic reprogramming, as discussed in Table 3.

### 4.1 Enhancing ferroptosis in CRC via NO modulation

The interplay between NO signaling and ferroptosis in CRC has garnered significant attention, focusing on exploiting redox imbalances to induce iron-dependent cell death. Ferroptosis, characterized by lipid peroxide accumulation and GSH depletion, is modulated by NO through direct enzyme inhibition, iron metabolism regulation, and potentiation of immunogenic cell death (ICD). Various NO donors induce ferroptosis in CRC cells. Compounds such as NCX4040 deplete intracellular GSH and elevate lipid peroxidation markers, and their effects are significantly enhanced when combined with ferroptosis inducers such as erastin or RSL3 (35). Other agents, including coumarin–furoxan hybrids and phenylsulfonyl furoxan derivatives, suppress the expression of solute carrier family 7 member 11 (SLC7A11) and covalently inhibit glutathione peroxidase 4 (GPX4), sensitizing cells to lipid peroxidation and helping reverse multidrug resistance ((121); 177). Sensitivity to ferroptosis is also regulated by other key proteins. Acyl-CoA synthase long-chain family member 4 (ACSL4) expression correlates with susceptibility, while NO-driven upregulation of heme oxygenase-1 (HMOX1) increases labile iron pools, amplifying lipid peroxidation (122). Lipidomic profiling confirms that NO donors lead to a marked increase in peroxidized phosphatidylethanolamines (PEs), a hallmark of ferroptosis (123).

GPX4, the primary enzyme for detoxifying lipid hydroperoxides, is a central target in NO-mediated ferroptosis. NO can directly inhibit GPX4 activity through mechanisms such as S-nitrosylation (124). Consequently, NO donors synergize with GPX4 inhibitors such as RSL3 to elevate markers of lipid peroxidation, while the ferroptosis inhibitor ferrostatin-1 can restore GPX4 activity and reduce lipid ROS (179, 125). The role of NO is context-dependent; while it can be protective in some cell types under specific conditions, higher NO concentrations in CRC consistently deplete GSH and inhibit GPX4, promoting a pro-ferroptotic outcome (82). Furthermore, NO produced by iNOS in M1 macrophages can exacerbate lipid peroxidation and ferroptosis in co-cultured CRC cells (123).

Advanced strategies using nanoplatforms and combinatorial therapies have shown promise. Nanocarriers such as AZOSH or IS@ATF enable triggered or targeted NO release, leading to GSH depletion, ONOO^−^ generation, and significant tumor weight reduction in murine models (36, 126, 127). Integrating NO donors with other treatments enhances therapeutic efficacy. Combination with immunotherapy (e.g., anti-PD-1) promotes immunogenic ferroptosis by increasing the infiltration of CD8^+^ T-cells, while combination with chemotherapy (e.g., 5-fluorouracil) increases chemosensitization in CRC cells (35, 36). Plasma-activated Ringer’s lactate, which generates NO and RNS, also induces ferroptosis effectively (128). Despite these promising results, challenges remain, including off-target vascular toxicity and the risk of systemic iron overload (36, 127). Future studies must prioritize tumor-specific delivery systems and the use of biomarkers such as ACSL4 and GPX4 for patient stratification to translate these findings into clinical practice.

### 4.2 NO’s role in mitochondrial dysfunction and oxidative stress

The activity of mitochondrial nitric oxide synthase (mtNOS) is a key driver of mitochondrial dysfunction, oxidative stress, and progression in colorectal cancer (CRC). Research shows that mitochondrial oxidative damage is a critical feature of CRC, with markers such as TBARS and protein carbonyls being significantly elevated in tumor tissues. The function of the electron transport chain (ETC) is also compromised, as shown by the reduced activity of key mitochondrial enzymes. Notably, mtNOS activity significantly increases in advanced-stage CRC, which correlates directly with markers of oxidative damage. This suggests that NO and hydrogen peroxide produced by mtNOS act as diffusible “toxohormones,” promoting oxidative stress in nearby non-tumor tissues and thereby facilitating tumor progression. This problem is further exacerbated by an imbalance in the cell’s antioxidant defenses as the activity of Cu, Zn-superoxide dismutase (SOD) is markedly reduced in advanced tumors. These findings directly implicate mtNOS hyperactivity in driving mitochondrial problems in CRC (129).

This destructive interplay between NO, ROS, and mitochondria is not unique to CRC; it is a common mechanism in many other cancers. For instance, in liver cancer, the drug sorafenib was found to increase intracellular NO and superoxide, leading to the formation of the highly reactive molecule peroxynitrite (ONOO^−^). This caused a drastic reduction in oxygen consumption and severe mitochondrial damage, a process that mirrors mtNOS-driven disruption observed in CRC (130). Similarly, in melanoma, the overexpression of a protein called UT-B was shown to increase NO levels, leading to mitochondrial depolarization, a spike in ROS, and reduced cell viability. This effect could be reversed with an antioxidant, directly linking NO overproduction to the observed oxidative stress (131).

NO overproduction in the tumor the microenvironment also plays a crucial role in promoting cancer progression. The loss of a protein called caveolin-1 (Cav-1) in stromal fibroblasts can lead to NO overproduction and a metabolic shift toward aerobic glycolysis. This phenomenon, known as the “Reverse Warburg Effect,” causes these fibroblasts to secrete lactate, which is subsequently used by cancer cells to enhance their mitochondrial activity and support proliferation. This shows how NO and ROS signaling from stromal cells creates a pro-tumor environment, reinforcing the “toxohormone” concept, where signals from one cell type promote cancer growth in another (129, 132). Similar metabolic shifts driven by NO and ROS have also been observed in lung cancer cells (133).

Interestingly, scientists are now turning this destructive mechanism into a therapeutic strategy. Mitochondria-targeted nanoplatforms that are designed to generate both NO and ROS have shown great promise. In hepatocellular carcinoma models, this approach produced high levels of ONOO^−^ inside mitochondria, causing irreversible damage to the ETC, inhibiting ATP production, and triggering cancer cell death, which significantly reduced tumor volume (134). Similar results have been observed using photodynamic therapy in lung cancer and with combination drug treatments in breast cancer, where increased NO and ROS lead to mitochondrial stress, autophagy, and apoptosis (121, 135, 136). In summary, mtNOS upregulation in CRC is a central cause of mitochondrial damage, driving oxidative stress and weakening the cell’s defenses. This mechanism is conserved across many cancers and even extends to the tumor microenvironment. Targeting this interplay between NO and mitochondrial function is, therefore, a highly promising therapeutic avenue for CRC and other cancers.

### 4.3 NO-releasing agents in overcoming tumor hypoxia

**FIGURE 3:**
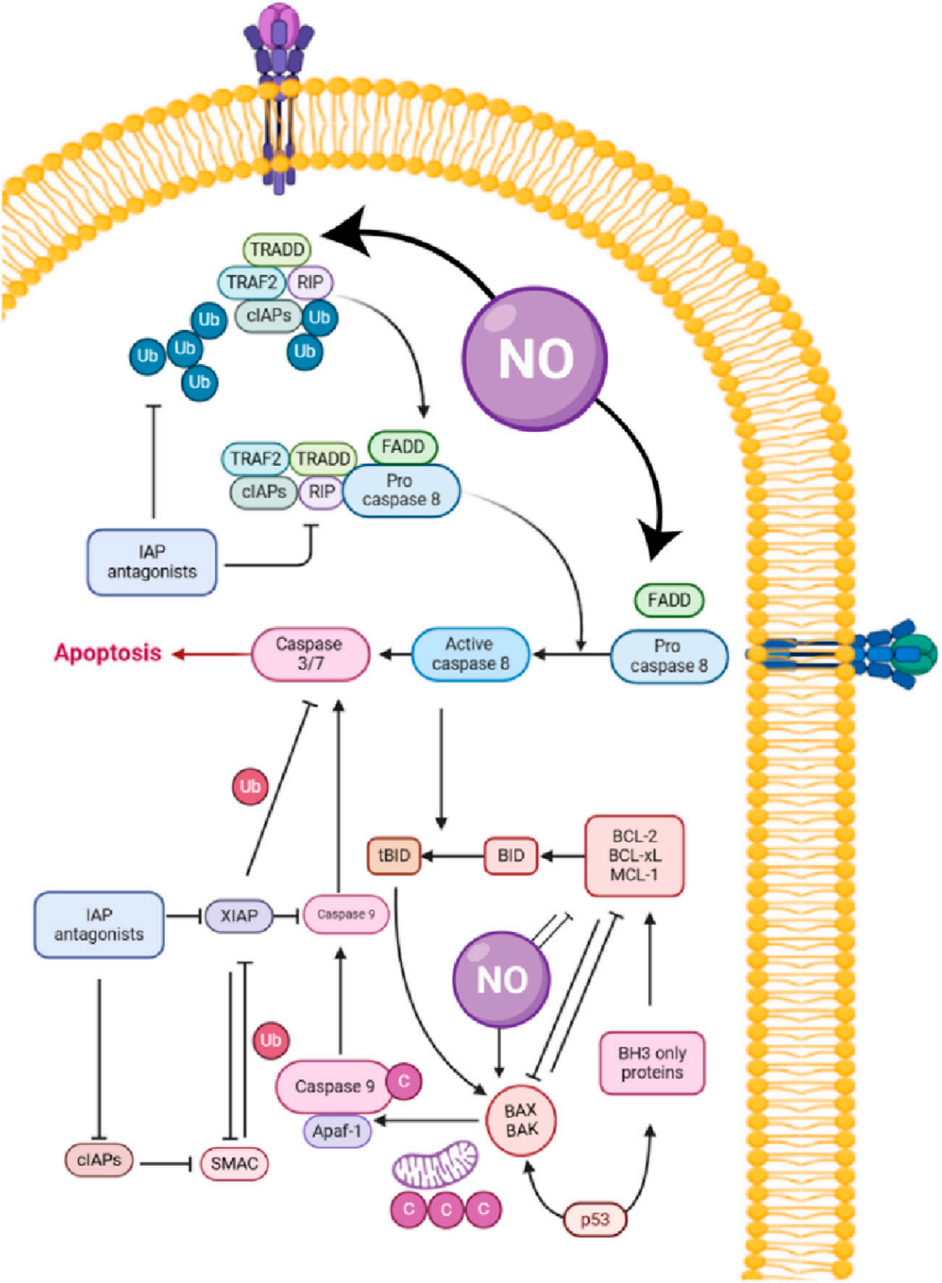
Role of nitric oxide (NO) in apoptosis pathways: mechanisms and interactions with cellular proteins.

Tumor hypoxia, a hallmark of solid malignancies such as CRC, drives therapeutic resistance, immunosuppression, and metastasis. NO, a gaseous signaling molecule, has emerged as a promising agent for modulating hypoxic microenvironments (Table 4). This discussion synthesizes findings from preclinical studies on NO-releasing strategies, emphasizing their applicability to CRC. Tu et al. (137) demonstrated that a micellar NO donor (TPGS-NO) enhanced radiotherapy efficacy in hypoxic tumors by improving oxygenation and reducing HIF-1α expression. TPGS-NO prolonged NO release in tumors, leading to increased angiogenesis and apoptosis (Figure 3) while inhibiting DNA repair post-radiation. Although tested in a non-CRC model, this mechanism is highly relevant to CRC, where hypoxia-driven resistance limits radiotherapy outcomes (137). Similarly, Dou et al. (138) developed radiation-activated nanoagents (NSC@SiO_2_-SNO NPs) that release NO upon X-ray irradiation. These nanoparticles reduced hypoxia and improved tumor oxygenation, monitored via BOLD/DWI imaging. This approach significantly inhibited tumor growth *in vivo*, suggesting potential for CRC applications where hypoxia compromises radiation efficacy (138). Zhang et al. (139) utilized ultrasound-stimulated microbubbles (USMBs) to enhance tumor perfusion and NO release in the MC38 murine colon cancer model. At a mechanical index (MI) of 0.3–0.5, USMBs increased tissue oxygen partial pressure (pO_2_) and reduced HIF-1α and lactate levels. Repeated treatments sustained hypoxia alleviation without resistance, highlighting a translatable strategy for CRC (139).

**TABLE 4:** Comprehensive analysis of nitric oxide-releasing agents in overcoming tumor hypoxia.

NO-releasing agent/intervention	Cancer model	Key finding	Mechanism	Reference
TPGS-NO micelles	General tumor hypoxia	Sustained NO release enhanced radiotherapy effects under hypoxia. Increased tumor radiosensitivity via improved angiogenesis and reduced hypoxia.	NO release improved tumor oxygenation and induced apoptosis.	(137)
Endogenous NO metabolites	Cutaneous melanoma patients	Serum NO metabolites were higher in melanoma patients than in healthy subjects. NO regulated hypoxia-inducible factors and immune suppression.	HIFs controlled tumor adaptation to hypoxia; NO modulated the immune microenvironment.	(144)
CTP/CDDP micelles (cisplatin + NO)	Hypoxic cancer cells	Reduced cisplatin efflux and inhibited EMT. Downregulated hypoxia-related pathways and enhanced anti-tumor effects.	pH-responsive NO release and targeted delivery.	(8)
Ultrasound + microbubbles (USMBs)	Colon cancer (mice)	Increased NO concentration and reduced hypoxia markers. Improved tumor perfusion and oxygenation.	Sononeoperfusion effect via NO release and eNOS activation.	(139)
NSC@SiO_2_-SNO nanoparticles	Hypoxic tumors	NO release under radiation improved tumor oxygenation. Imaging quantified oxygen levels and radiosensitivity.	Radiation-activated NO release enhanced hypoxia alleviation.	(138)
Endogenous NO	Tumor microenvironment	At low concentrations, NO promoted immunosuppression; at high concentrations, NO induced metabolic reprogramming.	Dose-dependent NO effects on tumor stroma and immune cells.	(145)
Supramolecular NO depot	Melanoma (mice)	Dual-phase NO release normalized tumor vessels and increased radiosensitivity. Synergistic effect reduced tumor growth.	NO-mediated vessel normalization and DNA damage fixation.	(146)
DETANONOate (NO donor)	Ewing sarcoma cells	NO inhibited mitochondrial O_2_ consumption under hypoxia. Glutamine depletion suppressed HIF stabilization.	NO modulated the mitochondrial response to hypoxia via glutamine metabolism.	(147)
PEG-PAMAM-PA/SNO nanoparticles	Hypoxic tumors	NO release depleted GSH and relieved hypoxia. Enhanced PDT efficacy.	GSH-responsive NO release combined with PDT.	(148)
P1-CapNO NPs (NIR-triggered)	Hypoxic tumors	NO release under NIR improved anti-tumor effects. Synergistic photothermal/NO therapy inhibited tumor growth.	Thermal-sensitive NO release and photothermal conversion.	(149)
RRx-001 (NO superagonist)	Tumor hypoxia	Enhanced NO generation from nitrite reduction reduced tumor resistance.	NO synthase-independent nitrite reductase activity.	(150)
IFN-γ + NO	Hepatoma cells	NO increased glycolysis and lactate production. HIF stabilization under hypoxia-regulated immune response.	NO modulated mitochondrial metabolism and HIF-1α.	(151)

Zhao et al. (140) combined ultrasound-targeted microbubble destruction (UTMD) with NO-generating nanodroplets (L-Arg@PTX). UTMD-triggered NO release reversed hypoxia, reduced cisplatin efflux, and enhanced cytotoxic T-lymphocyte infiltration. This dual approach improved chemoimmunotherapy outcomes, underscoring NO’s role in overcoming CRC immunosuppression (140). Chen et al. (141) designed chitosan-coated micelles (CTP/CDDP) co-delivering cisplatin and NO. In hypoxic cancer cells, NO downregulated HIF-1α, GSH, and multidrug resistance-associated protein 2 (MRP2), reversing cisplatin resistance. Although these findings were observed in non-CRC models, they are highly relevant to CRC, where hypoxia-driven chemoresistance remains a significant challenge (141).

Hypoxia stabilizes HIF-1α, promoting CRC progression. NO disrupts this pathway by inhibiting HIF-1α accumulation, as shown by Graham et al. (142), where NO/cGMP signaling blocked hypoxia-induced immune escape mechanisms. In CRC, this could enhance NK cell-mediated lysis by preserving surface MICA expression (142). This has direct implications for immunotherapy as hypoxia is known to increase PD-L1 expression via HIF-1α, enabling immune escape. As demonstrated by Barsoum et al. (143), NO donors such as nitroglycerin can attenuate this PD-L1 upregulation, thereby restoring T-cell cytotoxicity. This mechanism provides a strong rationale for combining NO-based therapies with checkpoint inhibitors such as anti-PD-1/PD-L1 to overcome hypoxia-induced immune resistance in CRC (143).

Although preclinical data are promising, clinical translation requires addressing NO’s biphasic effects; low doses alleviate hypoxia, whereas high doses may promote metastasis. Targeted delivery systems, such as CRC-specific nanoparticles or ultrasound-responsive agents, could mitigate off-target effects. Furthermore, there is a clear need for studies in CRC-specific models to optimize dosing and delivery schedules to ensure clinical viability. Additionally, combining NO donors with immunotherapy or hypoxia-activated prodrugs may amplify efficacy in CRC. NO-releasing agents represent a multifaceted strategy to combat CRC hypoxia. By enhancing perfusion, downregulating HIF-1α, reversing chemoresistance, and modulating immunity, NO synergizes with radiotherapy, chemotherapy, and immunotherapy. Further studies in CRC-specific models are warranted to optimize dosing and delivery, ensuring clinical viability.

### 4.4 Oxicam analogs and modulation of the NO pathway

Oxicam analogs exhibit novel capabilities in modulating the NO pathway offering unprecedented therapeutic potential. Unlike traditional anti-inflammatory agents, these compounds uniquely influence NO-related mechanisms, making them a promising frontier in treating inflammation and cancer (152). Krzystek-Korpacka et al. (153) and Dowling et. al. (154) highlighted the metabolic reprogramming in CRC, which includes the overexpression of enzymes such as ARG1, PRMTs, and DDAHs, in addition to NOS2. In the context of CRC, the NO pathway metabolites are found to be altered, with enzymes such as ARG1, PRMT1, and PRMT5 being overexpressed in both tumor and tumor-adjacent tissues. Notably, DDAH2 is overexpressed solely in tumor-adjacent tissue. The expression of ARG1 in tumors has been observed to increase with tumor grade and reflects lymph node involvement, indicating a potential role in disease progression (153, 154).

The modulation of this pathway by oxicam analog presents a promising therapeutic role (154, 155). Classic and novel oxicam analogs have been assessed for their impact on enzyme expression and intracellular metabolite concentration in CRC cell lines such as Caco-2, HCT116, and HT-29 (156). Novel oxicam analogs, particularly those with an arylpiperazine moiety at the thiazine ring, have shown greater efficacy in downregulating DDAHs and PRMTs and upregulating ARG2 compared to traditional oxicams such as piroxicam and meloxicam (157, 158). Oxicam derivatives significantly impact macrophage-associated chemokine expression, which is crucial in colorectal cancer pathophysiology. Their work suggested that these derivatives not only modulate the NO pathway but also exhibit dual COX-1/COX-2 inhibition, amplifying their anti-inflammatory properties (157). Similarly, Szczuka et al. (159) explored the interplay between oxicam compounds and heat shock proteins (HSPA1 and HSP90AA1), demonstrating potential therapeutic targeting in colorectal polyps and other malignancies (159). Moreover, (180) demonstrated the synergistic cytotoxic effects of oxicam derivatives with simvastatin, showing apoptosis induction in drug-resistant colon cancer cells (160).

In experimental models, Abdul Wanees El-Awdan et al. (161) tested combinations of meloxicam with octreotide, observing improved anti-inflammatory outcomes mediated through NO-dependent pathways (161). Research into stable lipoxin analogs underscores the connection between NO modulation and broader anti-inflammatory mechanisms, offering insights into drug development strategies (162). These studies collectively underscore the importance of oxicam analogs in NO pathway modulation, providing robust frameworks for clinical application in inflammatory disorders and oncology. These findings further suggest that metabolic reprogramming in CRC is not limited to tumor tissue and can be affected by novel oxicam analogs. This may provide a potential strategy for chemoprevention and therapy (Table 5).

**TABLE 5:** Recent advances in therapeutic compounds targeting nitric oxide and related pathways in colorectal cancer (CRC).

Compound/intervention	Reported target(s)/model	Mechanism(s) reported (concise)	Proteins/genes modulated (reported)	Hallmark/phenotype affected	Cells/models used	Combination(s) reported	Therapeutic implication (as stated by authors)	Pathway/mode	Key reference
NCX4040	Human CRC cells (HT-29, K-RAS mutant HCT-116)	Induces ROS, lipid peroxidation → ferroptosis-like cell death; ferroptosis inhibitor ferrostatin-1 protects cells	Upregulates/modulates CHAC1, GPX4, and NOX4 (transcriptomic/RT-PCR evidence); COX2/VEGF also changed in some contexts	Ferroptosis/oxidative-stress mediated death	HT-29 and HCT-116 (*in vitro*)	Ferroptosis inducers, such as erastin and RSL3, enhanced cytotoxicity; ferrostatin-1 inhibited NCX4040 cytotoxicity	NCX4040 kills CRC cells via ROS/ferroptosis-related mechanisms potential therapeutic lead, especially against Ras-mutant CRC in preclinical work.	Oxidative stress/lipid peroxidation/ferroptosis.	(35)
Nitric oxide (*ex vivo* human tissue study)	Human mesenteric arteries from CRC patients	Vasodilation via activation of vascular K+ channels; interacts with H_2_S signaling	Not a classical “protein inhibitor” functional activation of K^+^ channels (K_{ATP}, K_{Ca}, K_V, K_{ir}); measured effects on serum endocan/MDA	Endothelial/vascular function (vasodilation)	Human mesenteric artery segments (CRC patients) + blood serum measures	(Physiological/*ex vivo* study)	Demonstrates that NO- (and H_2_S-) mediated vasodilation occurs in mesenteric arteries supplying CRC and that gasotransmitter signaling is relevant in CRC patient vessels; serum endocan/MDA were measured as correlates.	cGMP/K^+^ channel-mediated vasodilation; cross-talk with H_2_S.	(118)
Hydrogen sulfide (same study as above)	Human mesenteric arteries (CRC patients)	Vasodilation via activation of K^+^ channels (notably, K_{ATP}, K_V types) and interaction with NO	Functional activation of K^+^ channels; study measured serum endocan and MDA	Endothelial/vascular function	Human mesenteric artery segments (CRC patients)		H_2_S contributes to vasodilation in mesenteric arteries of CRC patients and interacts with NO signaling, highlighting its potential relevance for tumor vascular biology.	K^+^ channel activation (K_{ATP}, K_V) and gasotransmitter cross-talk.	(118)
ZnPc-2NO, ZnPc-4NO (NO-releasing zinc phthalocyanines; PDT sensitizers)	*In vitro* cancer cell models (e.g., HT-29 colorectal and A549 lung demonstrated)	Release NO intracellularly; inhibit mitochondrial respiration → spare intracellular O_2_ for PDT → increase ROS during PDT; induce ICD features	Decreased ATP/mitochondrial respiration, reduced HIF-1α activity (lower oxygen availability), and ROS levels increased	Tumor hypoxia relief, enhanced PDT efficacy, and immunogenic cell death	HT-29 and A549 (cells) and PDT models (*in* *vitro*/*in vivo* contexts in the study)	PDT (light irradiation) no extra small-molecule combo reported as required	NO-releasing photosensitizers reduce O_2_ consumption by mitochondria, improve PDT under hypoxia, and promote ICD attractive strategy to overcome hypoxic resistance.	Respiration inhibition → increased ROS during PDT; HIF-1α downregulation mentioned.	(181)
Oxicam class compounds (including novel oxicam analogs and classic agents such as meloxicam/piroxicam)	Tissue and CRC cell lines (HCT-116, HT-29, and Caco-2); human CRC tissues profiled	Modulated L-arginine/NO metabolic enzyme expression (transcriptome and metabolome measured) can up/downregulate ARG2, DDAH1/2, NOS2, and PRMT1/5 depending on the compound	Reported changes in ARG2, DDAH1, DDAH2, NOS2, PRMT1, and PRMT5 (qPCR + metabolomics)	Tumor metabolic reprogramming; arginine/NO pathway alterations	Human CRC tissue samples (55 paired samples) and CRC cell lines HCT-116, HT-29, and Caco-2	Study evaluated drug effects *in vitro* (no clinical combination therapy tested in that paper)	Authors conclude that oxicam analogs modulate arginine/NO metabolism in CRC and are worth further study as potential anticancer agents that alter tumor arginine/NO homeostasis.	L-arginine/NO metabolic pathway (ARG, DDAH, and PRMT axis).	(153)
GSNO (S-nitrosoglutathione)	Proteomic S-nitrosylation analysis in CRC tissues and SW480 cells	GSNO/S-nitrosylation profiling (biotin-switch + MS) identifies endogenous and potential S-nitrosylated proteins related to metabolism, apoptosis, and inflammation	Recurrent S-nitrosylated proteins identified: ACTB (actin), PRDX4, PKM, GAPDH, ANXA4, and S100A8 (found in both human CRC tissue and SW480)	Modulation of metabolic and apoptotic signaling; potential biomarkers	Human CRC tissues and SW480 CRC cell line (proteomics analysis)	(Study is observational proteomics)	Authors highlight S-nitrosylation of specific proteins in CRC and propose SNO-proteins as contributors to CRC biology and potential early biomarkers.	S-nitrosylation (post-translational modification) affecting metabolic and apoptotic pathways.	(163)
Microcystin-LR (MC-LR)	SW480 CRC cells (*in vitro*)	Induces NO production → S-nitrosylation of GAPDH → GAPDH binds Siah1 and translocates to the nucleus → apoptosis (SNO-GAPDH–Siah1 cascade)	GAPDH and Siah1 pathway implicated (NOS involvement in SNO formation)	Apoptosis (NO/S-nitrosylation-mediated)	SW480 cells (*in vitro*)	GAPDH or Siah1 knockdown and NOS inhibition (L-NAME) attenuate apoptosis (mechanistic proof)	Demonstrates that MC-LR can trigger apoptosis in SW480 via a NO/S-nitrosylation cascade mechanistic insight into MC-LR cytotoxicity in CRC cells (note: MC-LR is a toxin; findings illuminate the underlying mechanism rather than proposing a therapy).	S-nitrosylation → GAPDH/Siah1 nuclear apoptosis pathway.	(52)
Apatinib (with piperine studied *in vitro*)	HCT-116 CRC cells (*in vitro* assay)	Apatinib reduces cell viability partly via downregulation of MDM2; observed changes in GPX (glutathione peroxidase) activity and increased NO levels in treated cells	MDM2 expression decreased (qPCR); NO levels and GPX activity measured as altered	Proliferation inhibition/apoptosis markers	HCT-116 cells (*in vitro*)	Piperine (co-treatment in the study enhanced effects)	Combination of apatinib + piperine showed synergistic cytotoxicity in HCT-116 *in vitro*, with concomitant MDM2 downregulation, and changes in NO/GPX suggest possible adjuvant strategies to increase cytotoxicity.	MDM2 regulation; oxidative stress/NO measurement as part of mechanism readouts.	(164)
Olaparib (metronomic/low dose)	Myeloid-derived suppressor cells and CRC immunotherapy models (mouse + *in vitro* MDSC assays)	Low (metronomic) PARP inhibition impairs MDSC suppressive function (reduces ARG-1/COX-2/iNOS expression) and synergizes with anti-PD-1 immune checkpoint therapy	ARG-1, iNOS, and COX-2 expression in MDSCs decreased after metronomic PARP inhibition; PARP-1 implicated	Immune suppression (MDSC function)/immune evasion	Tumor-derived MDSCs (mouse models) and preclinical CRC models (synergy with anti-PD-1 shown in mice)	Anti-PD-1 immunotherapy (synergistic in preclinical models)	Metronomic olaparib reduces MDSC immunosuppressive function and strongly enhances anti-PD-1 efficacy in preclinical CRC models (authors suggest repurposing low-dose PARPi to improve ICI responses).	PARP-1 inhibition → modulation of MDSC suppressive machinery; immune microenvironment remodeling (STING found to be dispensable for some effects).	(74)
Talazoparib	Tumor cells in preclinical cancer models; BRCA-deficient contexts emphasized	Potent PARP inhibition with PARP-trapping → DNA damage (γH2AX), activation of innate immune signals (STING, IRF3) at some doses; immune pathway upregulation	Markers of DNA damage (γH2AX) and STING/type I IFN pathway activation (IRF3, etc.) reported in tumor cells after PARPi	DNA damage response; immune activation (type I IFN/STING)	Tumor cell lines/BRCA-deficient tumor models; preclinical studies	Often evaluated in combination with ICI or other agents in preclinical studies	Talazoparib induces DNA damage and can upregulate immune pathways including STING/type I IFN in tumor cells rationale for combinations with ICI, especially in HR-deficient tumors; effectiveness associated with context (BRCA/HR defects).	PARP-inhibition → DNA damage → cGAS-STING/type I IFN immune signaling (dose- and context- dependent).	(165)
Hydrogen peroxide (H_2_O_2_, exogenous/ROS driver)	Cellular/epithelial models (general CRC/epithelial literature)	Oxidative damage to DNA and proteins; impairs detoxification systems (peroxiredoxins, GPX) and activates stress kinases and DNA damage checkpoints	Detox enzymes referenced widely in the literature: peroxiredoxins, GPX, catalase, etc. (studies show PRDX/GPX roles in H_2_O_2_ detoxification)	DNA damage response/oxidative stress/inflammation	Epithelial cell models; many CRC-related studies use H_2_O_2_ to model oxidative stress (reviewed work)	(Used as an experimental agent or pathophysiologic mediator)	H_2_O_2_ is a driver of oxidative DNA damage and pro-tumorigenic inflammation in colonic epithelia; detox systems (GPX and PRDX) moderate effect relevance as an oxidative-stress biomarker and mechanistic contributor to CRC.	Oxidative stress/DNA damage response (ROS signaling).	(166)
Iron (Fe^2+^/Fe^3+^; iron metabolism)	CRC biology literature and reviews	Dysregulated iron uptake and handling promote oxidative stress, support proliferation, and influence ferroptosis susceptibility; tumor cells often upregulate iron import (TfR1 and DMT1)	Iron-handling proteins and transporters reported in CRC reviews (e.g., TfR1, DMT1, and ferroportin changes described)	Proliferation/inflammation/ferroptosis sensitivity	CRC tissues and cellular models across multiple studies (reviewed)		Multiple reviews conclude that iron dysregulation (dietary heme/iron, increased uptake) contributes to CRC risk and tumor progression; iron metabolism is a therapeutic target (and influences ferroptosis-based strategies).	Iron metabolism/ferroptosis/ROS biology in CRC.	(167)
PG/ICG@MP (engineered microparticles delivering PG (NO donor) + ICG)	CRC cell lines and mouse models; LoVo, SW480, and MC38 mentioned in experiments	Particles deliver PG to tumor → generate endogenous NO and combine with ICG phototherapy under 808 nm irradiation → shift TAMs toward M1 (ARG1 inhibition and iNOS upregulation), boost ROS/NO synergy → antitumor immunity	Decreased ARG1 and increased iNOS in tumors/TAMs measured (WB/qPCR)	Tumor immune evasion/macrophage repolarization → promotes antitumor immunity	LoVo, SW480 (human), and MC38 (murine) and *in vivo* mouse tumor models	808 nm laser irradiation (phototherapy conditions)	PG/ICG@MPs modulate arginine metabolism (ARG1↓ and iNOS↑), reprogram TAMs away from M2, and enhance antitumor immune responses in CRC models translationally, promising nanotherapy by combining NO and phototherapy.	Arginine metabolism (ARG1/iNOS), NO and ROS synergy; TAM repolarization.	(73)
iNOS knockdown/iNOS downregulation (functional study)	SW480 CRC cells (*in vitro*) + *in vivo* metastasis readouts in referenced study	iNOS knockdown reduced NO and paradoxically promoted epithelial–mesenchymal transition (EMT) via a iNOS → GATA4 → CRB2 → E-cadherin regulatory axis (relocalization of E-cadherin)	GATA4, CRB2, and E-cadherin (relocalization rather than mRNA loss) transcriptomic changes associated with EMT shown	EMT/invasion/metastasis increased after iNOS downregulation in the study	SW480 cells (stable iNOS knockdown) and *in vivo* metastasis assays	The paper used iNOS inhibitor L-NIL and NO donor SNAP in mechanistic rescue experiments (context in the study)	The authors advise that lowering iNOS/NO can promote EMT and metastasis in some CRC contexts, indicating complex context-dependent roles for iNOS in CRC progression.	iNOS/NO signaling intersects with GATA4 and cell polarity/adhesion pathways → affects EMT.	(168)

## 5 Therapeutic implications and future perspectives in NO-Targeted colorectal cancer treatments

Emerging therapeutics target NO pathways to address these complexities. EV-CAT1, which inhibits arginine transport via the CAT1 transporter, not only suppresses NO-related angiogenesis but also enhances diagnostic accuracy when combined with carcinoembryonic antigen (CEA). Similarly, compounds such as NAD(P)H stimulate NO synthesis, identifying aggressive angiogenic phenotypes through cGMP-PKG signaling and providing avenues for therapeutic intervention. Furthermore, the interplay of NO and H_2_S in vascular modulation, particularly through agents such as SNP and Na_2_S, offers potential in regulating endothelial dysfunction and oxidative stress. These compounds activate K^+^ channels (e.g., KATP and KV), revealing a novel strategy to manage CRC progression. Future directions highlight integrating genetic insights and tumor microenvironment dynamics into precision therapies. Combining NO pathway modulators with innovative diagnostic tools, such as EV-CAT1, could significantly improve treatment efficacy. The synergistic targeting of NO and gasotransmitter pathways, such as H_2_S, represents an exciting frontier in developing robust anti-cancer strategies for CRC.

Therapeutic strategies targeting NO have also shown promise in preclinical models and present potential for CRC treatment (Table 6). NCX4040, targeting CHAC1 and GPX4, induces ferroptosis in Ras-mutated CRC cells, suggesting future applications in oxidative stress-focused therapies. ZnPc-2NO and ZnPc-4NO inhibit mitochondrial respiration, reducing oxygen consumption and offering strategies against hypoxic tumors via ICD. Modulating ARG1 and iNOS polarizes TAMs from M2 to M1 phenotypes, enhancing immune responses and positioning ARG1 as a key therapeutic target for macrophage-directed interventions (181).

**TABLE 6:** Experimental and clinical findings on NOS-targeting agents in colorectal cancer research.

Drug/compound/item	Pathway targeted	Model/study type (as reported)	NOS/NO interaction (as reported)	Key experimental finding(s) (accurate, not paraphrased beyond the paper)	Clinical significance/interpretation (as reported/authors’ conclusion)	Reference
Celastrol	Angiogenesis and NOS (iNOS/eNOS)	*In vitro*—HT-29 and HCT116 cells; mechanistic assays	Celastrol inhibited NOS activity (iNOS and eNOS) in colorectal cancer cells	Celastrol inhibited proliferation and migration of HT-29 and HCT116 cells; effects associated with the inhibition of iNOS/eNOS and angiogenesis pathway components.	Authors conclude that celastrol suppresses CRC cell growth/migration partly via suppression of NOS and angiogenesis pathways (possible chemo-sensitization implications).	(169)
NOS inhibitors (1,400 W and L-NIO)	Angiogenesis pathway	*In vitro* colorectal cancer cell lines (same research group assays)	1,400 W (iNOS inhibitor) and L-NIO (eNOS inhibitor) inhibit NOS activity	Treatment reduced markers of angiogenesis-related signaling and suppressed colorectal cancer cell growth and migration *in vitro*.	Authors reported that NOS inhibitors suppressed CRC cell growth/migration, likely via angiogenesis pathway suppression—suggesting NOS blockade as therapeutic strategy.	(170)
eNOS polymorphisms (894G>T)	eNOS/metabolic-syndrome interaction	Human clinical cohort (CRC patients; analysis of eNOS SNPs and outcomes)	The eNOS 894G>T polymorphism was examined in relation to outcomes in CRC patients with MetS	The study reports that eNOS 894G>T (in interaction with the metabolic syndrome status) was associated with poorer clinical outcome; the eNOS polymorphisms alone were not associated with MetS prevalence.	The authors concluded that eNOS 894G>T combined with MetS is associated with worse prognosis in CRC—possible prognostic marker in that subgroup.	(171)
Quercetin	Anti-inflammatory and NO metabolism	*In vivo*: AOM/DSS-induced colon carcinogenesis mouse model	Quercetin reduced NO and oxidative stress-related markers in colon tissue (reported LPO, NO, and antioxidant enzyme changes)	Quercetin treatment significantly reduced the number and size of colon tumors, reduced inflammation and lipid peroxidation (LPO), and modulated NO and antioxidant markers in AOM/DSS mice.	Authors concluded that quercetin exerts chemopreventive effects in this CRC model via anti-inflammatory and antioxidant (including NO modulation) actions.	(172)
iNOS—correlation with VEGF/angiogenesis (68)	Tumor angiogenesis/VEGF	Human surgical tumor specimens (immunohistochemistry; 46 specimens reported)	iNOS expression correlates with VEGF expression and microvessel density (MVD) in human CRC tissue	Study found a strong correlation among iNOS immunostaining, VEGF expression, and MVD; higher iNOS was associated with increased angiogenesis markers.	Authors consider iNOS as a contributor to VEGF-mediated angiogenesis in human colorectal cancer and its possible role in tumor progression.	(68)
Doxorubicin—NO, calreticulin, and phagocytosis	NO and immunogenic cell death	*In vitro*—HT-29 colon cancer cells (drug-sensitive vs. drug-resistant lines)	Doxorubicin did not induce NO synthesis and calreticulin (CRT) exposure in Dox-sensitive HT29 cells and HT29 iNOS	In drug-sensitive HT29 cells, doxorubicin did not induce NO production, CRT exposure, and cell phagocytosis; resistant cells lacked these responses.	Implication: NO induction contributes to immunogenic cell death elicited by doxorubicin in sensitive colon cancer cells; resistance is associated with loss of the NO/CRT response.	(173)
iNOS and COX-2	Angiogenesis (iNOS ↔ COX-2 cross-talk)	Human tumor samples and CRC cell lines (IHC and functional assays)	NO stimulates COX-2 pathway elements and vice versa; correlated expression in tumors	Study reports that COX-2 activity mediates pro-angiogenic effects and correlates with iNOS expression; NO can upregulate PGE2 and VEGF signaling.	Authors suggest that COX-2 activation contributes to NO-mediated angiogenesis; COX-2 inhibitors may modulate NO-driven pro-angiogenic effects.	(174, 175)

Olaparib’s capacity to suppress MDSCs and enhance T-cell function, particularly in combination with anti-PD-1 therapy, underscores its potential role in immunotherapy for MSS tumors. NOS inhibitors, such as 1,400 W and L-NIO, combined with 5-fluorouracil, enhance anti-CRC effects, paving the way for dual-pathway treatments. Celastrol’s and atorvastatin’s anti-tumor activities suggest potential as chemopreventive agents targeting NO signaling.

NO-releasing nanotechnologies, delivering localized therapeutic doses, minimize systemic toxicity and address CRC’s adaptive resistance mechanisms. These advancements align with future CRC treatments focusing on precise NO modulation and combination regimens to overcome resistance and optimize outcomes.

## 6 Conclusion

NO has emerged as a critical mediator in the complex biology of CRC, exhibiting a dual role as a tumor promoter and a tumor suppressor. This duality presents significant challenges and opportunities for therapeutic intervention. NO exerts diverse effects on CRC pathophysiology, including promoting ferroptosis, modulating the tumor immune microenvironment, driving metabolic reprogramming, and enhancing metastatic capacity. The complexity of NO signaling is shaped by its concentration, intracellular localization, and interactions with other molecular pathways, such as H_2_S, underscoring the importance of these contextual factors. Current research efforts focused on the precise modulation of NO or selective targeting of NO-related pathways represent a promising frontier for enhancing CRC treatment efficacy. These approaches hold the potential to overcome resistance mechanisms and enhance the effectiveness of existing therapies. Developing targeted therapies that leverage NO’s complex roles will require a more comprehensive understanding of the delicate balance between its tumor-promoting and tumor-suppressing activities. Achieving this understanding is essential for creating interventions that can strategically influence NO’s effects in CRC, potentially leading to more refined and effective treatment strategies.

## Glossary

APC: Adenomatous polyposis coli
ALOX15: Arachidonate 15-lipoxygenase
ADMA: Asymmetric dimethyl arginine
CHAC1: Cation transport regulator homolog 1
CTCs: Circulating tumor cells
CBS: Cystathionine β-synthase
CSE: Cystathionine γ-lyase
COX-2: Cyclooxygenase-2
DDAH1: Dimethylarginine dimethylaminohydrolase 1
eNOS: Endothelial nitric oxide synthase
EMT: Epithelial–mesenchymal transition
ERK: Extracellular signal-regulated kinases
EVs: Extracellular vesicles
GAPDH: Glyceraldehyde-3-phosphate dehydrogenase
GLS2: Glutaminase 2
GPX4: Glutathione peroxidase 4
CAT1: High-affinity cationic amino acid transporter 1
**H**_**2**_ **S**: Hydrogen sulfide
iNOS: Inducible nitric oxide synthase
IFN-γ: Interferon-gamma
IL-6: Interleukin-6
Substrate for nitric oxide synthesis: L-arginine
MAPK: Mitogen-activated protein kinases
MDSCs: Myeloid-derived suppressor cells
NOX4: NADPH oxidase 4
nNOS: Neuronal nitric oxide synthase
NO: Nitric oxide
NOS: Nitric oxide synthase
PPP: Pentose phosphate pathway
**ONOO** ^**−**^: Peroxynitrite
PDE2A: Phosphodiesterase 2A
PDE5A: Phosphodiesterase 5A
PRMT1: Protein arginine methyltransferase 1
PRMT5: Protein arginine methyltransferase 5
PKG: Protein kinase G
RNS: Reactive nitrogen species
ROS: Reactive oxygen species
SNO: S-nitrosylation
sGC: Soluble guanylate cyclase
TMEM180: Transmembrane protein 180
TAMs: Tumor-associated macrophages
TNF-α: Tumor necrosis factor-alpha
VEGF: Vascular endothelial growth factor
